# Comparison of two different lingual flap advancement techniques and vascular structure identification: a human cadaver study

**DOI:** 10.4317/medoral.25451

**Published:** 2022-10-16

**Authors:** Carlos Noguera-Mutlló, Bassel Traboulsi-Garet, Octavi Camps-Font, María Cristina Manzanares-Céspedes, Rui Figueiredo, Eduard Valmaseda-Castellón

**Affiliations:** 1 DDS, MS. Master of Oral Surgery and Implantology. Faculty of Medicine and Health Sciences, University of Barcelona, Barcelona, Spain; 2 DDS, MS. Master of Oral Surgery and Implantology degree program. Faculty of Medicine and Health Sciences, University of Barcelona, Barcelona, Spain; 3 DDS, MS, PhD. Master of Oral Surgery and Implantology. Associate Professor of Oral Surgery and Professor of the Master of Oral Surgery and Implantology degree program. Faculty of Medicine and Health Sciences, University of Barcelona, Barcelona, Spain.; 4 MD, PhD. Professor of Human Anatomy and Embryology, Faculty of Medicine and Health Sciences, University of Barcelona, Barcelona, Spain; 5 DDS, MS, PhD. Master of Oral Surgery and Implantology. Associate Professor of Oral Surgery and Professor of the Master of Oral Surgery and Implantology degree program. Faculty of Medicine and Health Sciences, University of Barcelona. Researcher at the IDIBELL Institute, Barcelona, Spain; 6 DDS, MS, PhD. Master of Oral Surgery and Implantology. Professor of Oral Surgery. Director of the Master of Oral Surgery and Implantology degree program. Faculty of Medicine and Health Sciences, University of Barcelona. Researcher at the IDIBELL Institute, Barcelona, Spain

## Abstract

**Background:**

One of the most frequent complications in guided bone regeneration (GBR) is wound dehiscence, which compromises treatment outcomes. Thus, primary tension-free suture is essential to avoid wound dehiscence. The purpose of this study was to compare the extension of 2 different mandibular flaps in human cadaveric specimens, and to measure the size of the supraperiosteal blood vessels.

**Material and Methods:**

Five freshly unfrozen human cadaveric specimens were used. Arteries and veins were marked and bilateral classical lingual flaps (extending from the second premolar to the retromolar area) were prepared. In one side, the mylohyoid muscle was detached to increase the coronal extension of the flap. An implant drill was used to measure the extension of the flap after exerting 30 g of traction, before and after detaching the mylohyoid muscle. The size of the largest vascular structures of the flap was measured using a periodontal probe.

**Results:**

The classical flap extension was 5.99 mm (95% confidence interval (CI): 5.08 to 6.90), while the coronally advanced flap extension with mylohyoid muscle detachment was 14.96 mm (95%CI: 10.81 – 19.11). A statistically significant difference was found between the 2 groups (*p*= 0.0002), with a mean extension difference was 8.97 mm (95%CI: 5.02 to 12.91). The mean largest artery had 0.20 mm of diameter (95%CI: 0.15 – 0.26).

**Conclusions:**

The detachment of the mylohyoid muscle from the lingual flap allows to significantly increase its extension by 2.5 times. The superficial arteries found in the lingual flap have a small diameter (around 0.2mm).

** Key words:**Alveolar bone grafting, bone regeneration, surgical wound dehiscence, dental implants, soft tissue management.

## Introduction

Implant treatment has dramatically changed in the last 50 years, since Brånemark first defined described the concept of osseointegration (1). As indications for implant therapy gradually increased, new techniques were proposed in order to ensure an adequate bone volume and achieve optimal results (2). Some of these techniques are guided bone regeneration (GBR), onlay or inlay autogenous bone blocks and split autogenous bone blocks (3-5).

Any technique of surgical ridge augmentation requires not only a complete closure of the wound without tension (6-7), but angiogenesis, space creation for bone growth, and stability (7). One of the crucial steps is primary wound closure, which can be jeopardized by subsequent edema. Wound dehiscence is one of the main threats to bone augmentation and can occur in 7 to 13% of the cases (8,9). Risk factors for this complication are, among others, suturing technique, suture material, and flap design (10-12).

Non-resorbable membranes are widely employed in vertical ridge augmentation procedures (13,14). These barriers are particularly susceptible to wound dehiscencies, because membrane exposure usually leads to contamination, thus compromising the final results (15). In vertical augmentations performed in the posterior area of the mandible, where soft tissues are thin, and flap borders need to be stretched, membrane exposure might be more common (14-16). Therefore, optimal soft tissue management is required to achieve a primary closure of the flap without tension and to prevent infection of the membrane and the underlying material (10,17).

Burkhardt *et al*. (10) reported a risk of dehiscence around 10% if flap tension before suturing was lower than 10 g. However, this Figure increased to 40-100% if tension exceeded 10 g, and all flaps sutured at more than 25.5 g had a dehiscence. A suture with low flap tension can be achieved by means of the combination of a suspended external-internal suture, which drastically reduces the tension at the flap border that is sutured with a second tension-free suture closer to the wound margin (18). The suture material and the adequate management of the wound using releasing incisions are of paramount importance as well (12).

**Figure 1:**
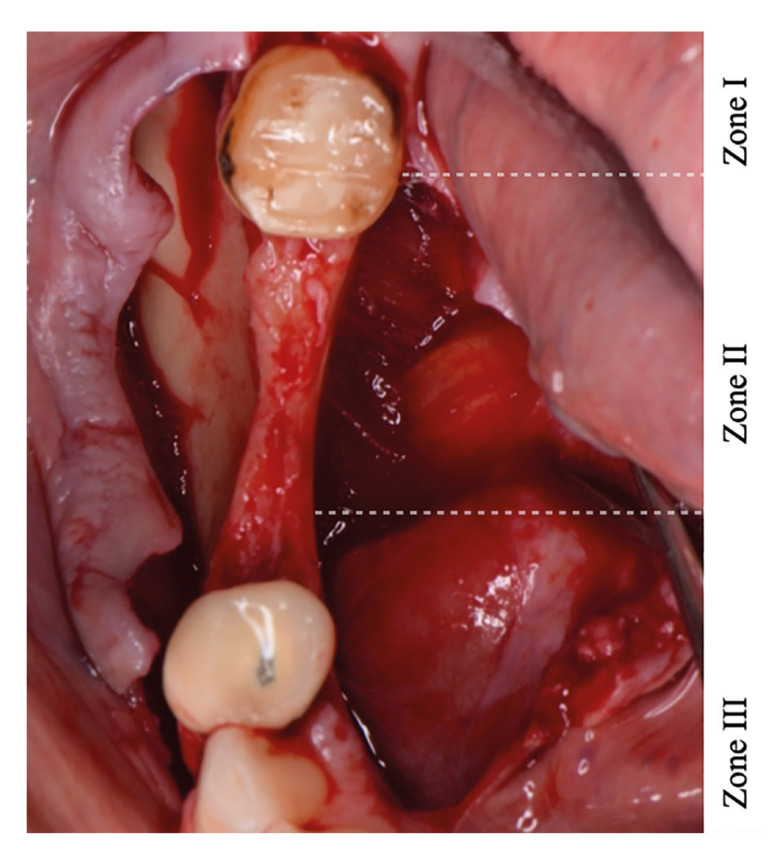
Flap design and location of zones I, II and III.

Several flap management techniques have been proposed to gain correct closure (19,20). Ronda *et al*. (14) proposed a technique to achieve coronal displacement of the lingual flap for posterior mandibular ridge augmentation. A full-thickness lingual flap is to be raised and partially detached from the mylohyoid muscle. Urban *et al*. (21) describes this technique, dividing the surgical approach of the lingual flap in 3 different zones (zone I: tunnelling and lifting of the retromolar pad, zone II: flap separation with mylohyoid muscle preservation, zone III: anterior, semiblunt periosteal release) (Fig. 1).

This approach provides an adequate advancement of the lingual flap which, in turn, facilitates a tension-free wound closure. Mobilization of the lingual flap is nowadays a requirement for vertical ridge augmentation in the mandible.

The lingual aspect of the mandible has been considered a danger zone due to the proximity of the lingual nerve and lingual blood vessels. Indeed, hematoma and severe bleeding have been reported during or after implant surgery in the mandible (16). The sublingual artery, a branch of the lingual artery which runs between the mylohyoid and the genioglossus muscle can give some small branches that supply the gingiva (16). Although terminal branches of the lingual artery might be found in the lingual flap, the lingual artery is not at risk, provided blunt dissection is performed. If bleeding results, usually it is the result of trauma to the superficial terminal branches over the periosteum (22).

The main aim of the present study was to confirm the findings described by Urban *et al*. (21) regarding the effect of detaching the mylohyoid muscle from the lingual flap on the flap extension in the posterior mandibular zone of human cadavers. A secondary objective was to measure the diameter of the superficial arterial branches of the lingual flap.

## Material and Methods

- Sample preparation: Washing and drying the cadaveric specimens

Five fresh frozen cadaver specimens, donated to the Dissection Room and Donation Service of the Faculty of Medicine and Health Sciences of the University of Barcelona, were used. The specimens were preserved at -16ºC since its admission in the dissection room, then, after being defrosted, orofacial arteries and veins were differentially marked. The first specimen was used to test the process and its measurements were not included in the analysis. The study was carried out with the approval of the Research Bioethics Committee from the University of Barcelona (IRB00003099).

A pump device was used for washing the craniofacial arterial and venous circuits, first with water and then with a mixture of embalming solution (phenol 90%: 12.5 mL; ethanol 96%: 62.5 mL; 35%-40% formaldehyde solution: 7.5 mL; and glycerol 17.5 mL). The solution was injected through the internal jugular veins and the common carotid artery with a pump device. A total of 5 L of the solution were injected into each specimen, in order to extract any rest of clots from the vascular structures. After at least 3 injections, the heads were positioned upright and manually drained. Finally, air was injected into both jugular and carotid vessels to facilitate the filling (23).

- Dissection and ligation of the carotid arteries

The carotid bifurcation was carefully dissected to identify the external and internal carotid arteries. The internal carotid artery was ligated with 3-0 silk sutures (silk suture 3/0, Silkam, B. Braun, Rubí, Spain). Ligatures prevented latex from invading the surrounding structures, while ensuring the filling of facial and lingual arteries and its branches. External carotid arteries and jugular veins were cannulated and sutured, which facilitated injection of a pressurised latex preparation through plastic canulae (14,22).

- Latex preparation and injection

Both external carotid arteries and both internal jugular veins were injected with latex (RV-30L, GELIF/F; Resinpol Latex Compound SL., Terrassa, Spain). Red latex was injected through the external carotid arteries and blue latex was injected through the internal jugular veins, as previously described by Caraballo *et al*. (23) Afterwards, the specimens were preserved 24 hours at a constant temperature of +5ºC to ensure latex gelling.

- Surgical procedure

A classical lingual flap was prepared (without detachment from the mylohyoid muscle) in both sides of the mandibles (control group, n=10) and its coronal extension was measured. Then, a coronally advanced lingual flap was prepared by dissecting the lingual flap from the mylohyoid muscle (test group, n=10) and its extension measured (16).

All procedures were performed by the same surgeon (CMN) under the same environmental conditions to control for technical consistency.

A full-thickness crestal incision was performed in the keratinized tissue from the retromolar area to the second premolar area. If premolars were present, a sulcular incision in the buccal side and in the second premolar in the lingual side was carried out. Finally, a buccal vertical releasing incision of 9 mm (beyond the mucogingival junction) was performed, and a lingual vertical releasing incision of 4 mm at the mesial side of the second premolar (14,22).

**Figure 2:**
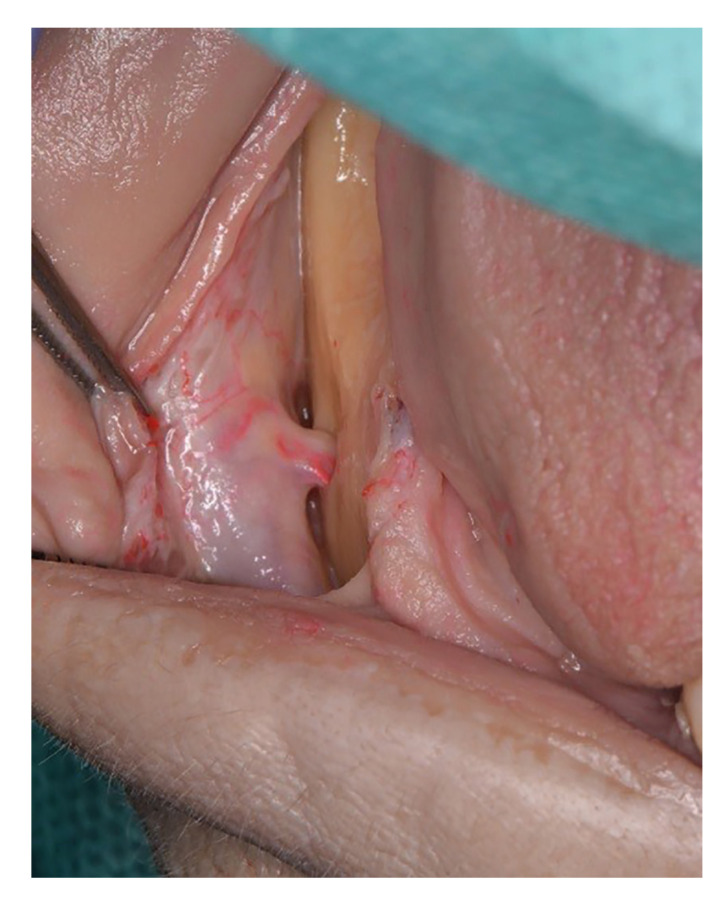
Identification of the mental nerve to ensure correct placement of the incision.

Then a full-thickness flap was raised both buccally and lingually. The mental nerve was identified to ensure that the buccal flap was extended to the mesial aspect of the mental nerve (Fig. 2).

**Figure 3:**
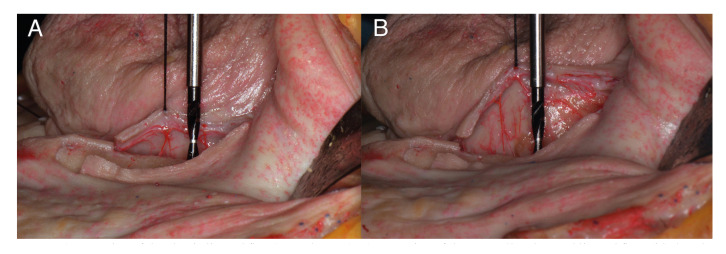
A) Extension of the classic lingual flap (control group); B) Extension of the coronally advanced lingual flap with detachment of the mylohyoid muscle (test group). The bur was used for calibration in the photographs.

A lingual flap was raised (control group), which consisted of a full-thickness flap at the lingual aspect of the alveolar ridge. After a careful soft tissue management, a silk suture was placed in the middle of the lingual flap (silk suture 3/0, Silkam, B Braun, Rubí, Spain). The lingual flap was coronally pulled with a force of 30 g (0.2942 N) using a pulley system which was previously calibrated with a dynamometer (Digitale Weegschaal 25 kg ± 10 g, Sensas, France). Then, the flap extension was photographed with a calibrated implant bur for reference (Fig. 3). Next, a coronally advanced lingual flap with detachment of the mylohyoid muscle was performed (test group), using the technique described by Urban *et al*. (22), which differentiates zones I, II and III, following the same methodology (Fig. 3).

The calibrated implant bur was placed 8 mm inside of the crestal bone at the middle of the exposed alveolar crest (drill Ø2 8-16 TPR, Nobel Biocare, Danaher Corp.®, USA) (Fig. 3). Photographs were obtained at the same distance, height and angulation by means of a tripod (Nikon D5100; AF-S Micro, 85 mm, 1:35 G, Nikon, Nikon Corp.®, Japan). Image J software (ImageJ 1.51k, Wayne Rasband, National Institutes of Health, USA) was used to calibrate pictures using the implant bur as reference and measure flap extensions from the crestal bone surface. Preparation of the flaps and measurements were carried out by the same investigator (CNM).

**Figure 4:**
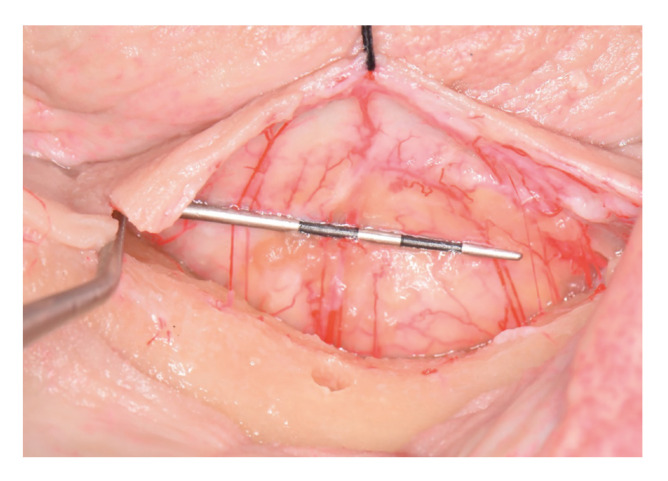
In each lingual flap a photography was performed to measure the artery with the largest diameter. A periodontal probe was used to calibrate the measurement.

Finally, another photograph was obtained in each side with a periodontal probe (Thin Williams Probe, Hu-Friedy Mfg. Co., LLC, Chicago, USA) as a reference. This calibrated image was used to measure the widest artery of the flap (Fig. 4).

- Statistical Analysis

The statistical analysis was performed using the Stata 14 statistical package (StataCorp®, College Station, USA). The normal distribution of continuous quantitative variables was assessed using the Shapiro-Wilk test (*p* > 0.10) and visual analysis of the normal P-P plot and box plots. The distributions did not differ from normality; hence the mean, standard deviation (SD), and 95% confidence intervals (95%CI) of all measurements were calculated. Differences in flap extension between test and control group and by gender were assessed with a paired t-test.

## Results

Mean age at death of the donors (3 women, 2 men) was 81.4 years (range 79 - 92; 95%CI: 73.5 - 89.3).

The mean flap extension in the control group was 5.99 mm (SD=1.27; 95%CI: 5.08 to 6.90), with a mean value of 6.27 mm (95%CI: 4.23 to 8.30) at the right side and 5.72 mm (95%CI: 4.63 to 6.80) at the left side. The mean flap extension of the test group was 14.96 mm (SD=5.80; 95%CI: 10.81 to 19.11), with a mean value of 14.83 mm (95%CI: 6.92 to 22.74) at the right side and 15.09 mm (95%CI: 7.74 to 22.46) at the left side (Table 1).

The coronally advanced lingual flap with detachment of the mylohyoid muscle achieved 2.5 times more extension than the classical lingual flap, with a mean difference of 8.97 mm (95%CI: 5.02 to 12.91), which was statistically significant (t= 4.77; df=18; *p*= 0.0002; Fig. 3).

There were no statistically significant differences between genders in terms of flap extension, although longer extensions were found in women [mean difference of 0.67 mm (95%CI -1.27 to 2.60) in the control group; and 3.51 mm (95%CI: -5.20 to 12.21) in the test group].

The mean vascular calibre was 0.20 mm (95%CI: 0.15 to 0.26), with a mean of 0.24 mm (95%CI: 0.12 to 0.37) for the right side and 0.17 mm (95%CI: 0.15 to 0.18) for the left side (Table 1, Fig. 4).

**Table 1:**
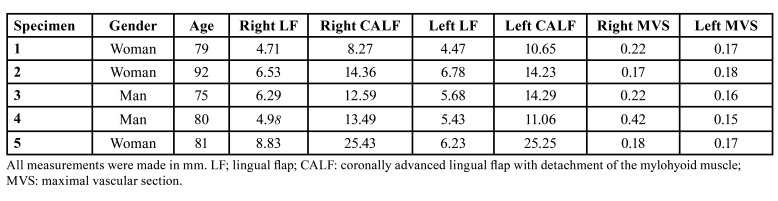
Measurements of the flap extensions and maximal vascular section.

Discusion

The proposed coronally advanced lingual flap showed a 2.5-fold extension in comparison with the classical advanced lingual flap used as control in this cadaveric study. Nevertheless, this research has some limitations. On one hand, freshly unfrozen cadaveric material might lose some elasticity (24). This might lead to an overestimation in the difference of flap extension. To reduce the effect of this bias, a cross-over design was used (both techniques were performed on each side). On the other hand, blinding was not possible due to the study design. However, the pulley system with the dynamometer and the standardized photographs ensured precision of measurements. Besides, to assure technical consistency, all procedures were performed by the same surgeon.

Our results are similar to those of Urban *et al*. (22), who achieved a difference between the baseline extension (an initial flap elevation) and the final extension (the coronally advanced lingual flap) of 9.27 mm (SD 2.19) in zone I; 16.45 mm (SD 2.876) in zone 2, and 12.63 mm (SD 2.87) in zone III. The present comparative study achieved a mean difference of 8.97 mm (SD=4.53), which was slightly lower, probably because the control group had a vertical releasing incision, which provided more initial extension and reduced the difference between both flaps.

As tension-free primary closure of the wound is required in augmentation techniques, adequate release of both buccal and lingual flaps is required (25,26). At the lingual aspect, care must be taken not to damage the lingual nerve, the sublingual artery, and the sublingual (Wharton’s) duct (13,27). These are not commonly found in a close relation to the periosteum of the lingual flap, except for the lingual nerve, which might be superficial near the molars, so blunt dissection is required in the so-called zone I (22,28). However, some anatomical variations might be present: in around 2/3 of cases, the sublingual artery supplies the sublingual space, while in almost 1/3 of cases there is no sublingual artery and a branch of the submental artery supplies the sublingual space. Submental and sublingual arteries anastomose and irrigate the floor of the mouth in 6% of the cases. In around 2% of cases, the submental artery supplies the floor of the mouth but both sublingual and deep lingual artery are missing. When the submental artery supplies the submental space (29.6%) the same artery perforates the mylohyoid muscle and enters the lingual space (16). Hence, these cases might pose a higher risk of vascular injury and bleeding, thus blunt dissection is mandatory (22).

In the present cadaveric study, blood vessels of approximately 0.20 mm (range 0.15 to 0.41 mm) were observed in the superficial surface of the lingual flap periosteum. These vessels are quite small when compared with the posterior superior alveolar artery, which might be injured during a sinus lift procedures (around 1 mm). So potential bleeding in these areas can be easily controlled with local measures in normal conditions (29). Although life-threatening haemorrhage has been reported after implant placement in the posterior mandible, it was associated with drilling and perforation of the lingual plate, which might sever branches of the lingual artery and mylohyoid muscle, as well as the lingual nerve (30).

As conclusions, the detachment of the mylohyoid muscle from the lingual flap allows to significantly increase its extension by 2.5 times. Thus, this manoeuvre might be indicated in bone augmentation procedures of the posterior mandible that required a tension-free closure. The superficial arteries found in the lingual flap have a small diameter (around 0.2mm).
